# Supplementary material for: Cross-Linking Mass Spectrometry on P-Glycoprotein
Source: Int J Mol Sci. 2023 Jun 25;24(13):10627. doi: 10.3390/ijms241310627 (PMC10341432; doi:10.3390/ijms241310627)
Supplement: Supplementary file 1 [file ijms-24-10627-s001.zip › ijms-2439873-supplementary.pdf]

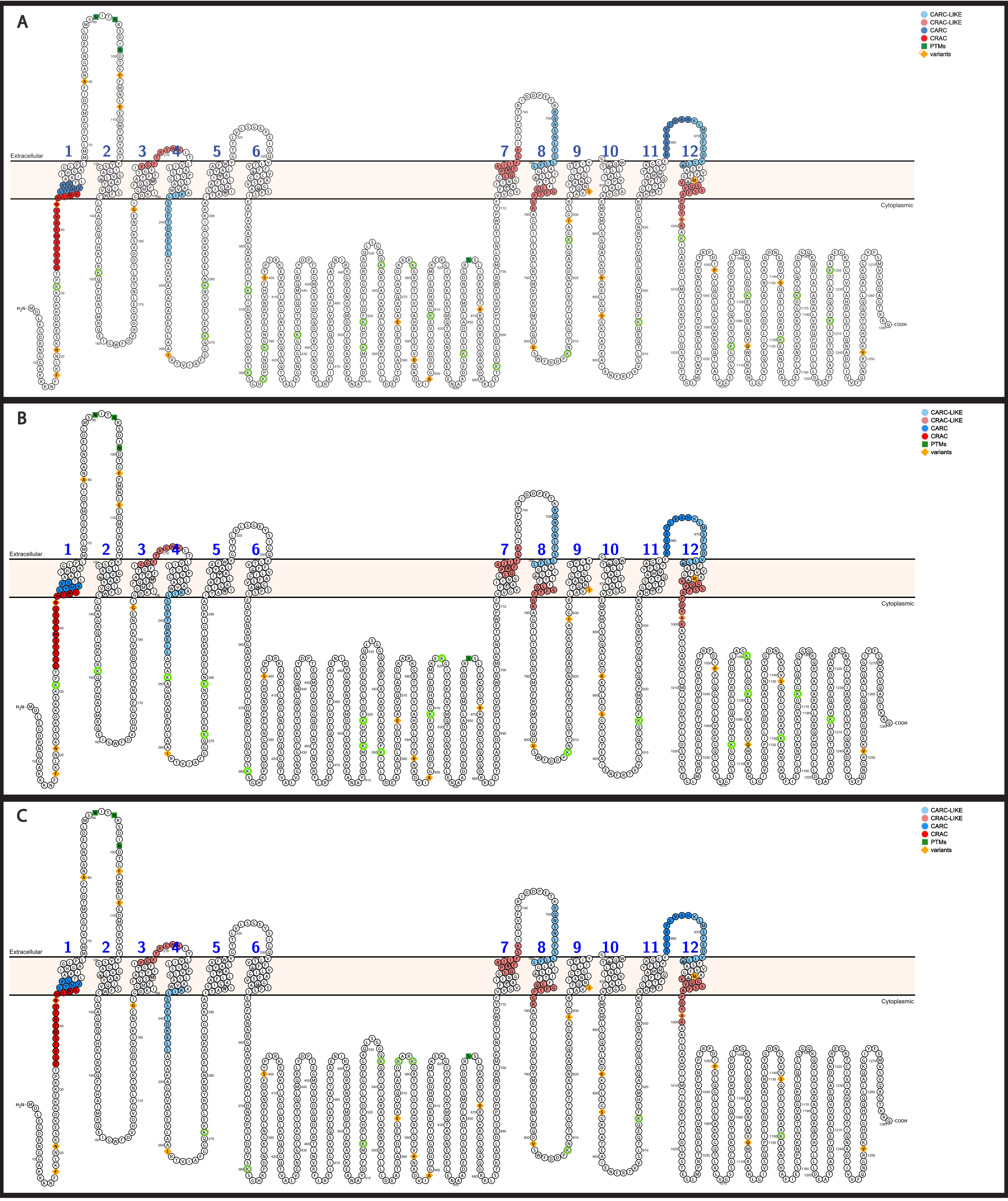

Figure S1. Mono-links of Pgp visualized on its primary structure by Protter 1.0. Mono-links on Pgp in the **(A)** on-bead DSSO, **(B)** the living cell DSSO and **(C)** living cell BS2Gd0/d4 samples.

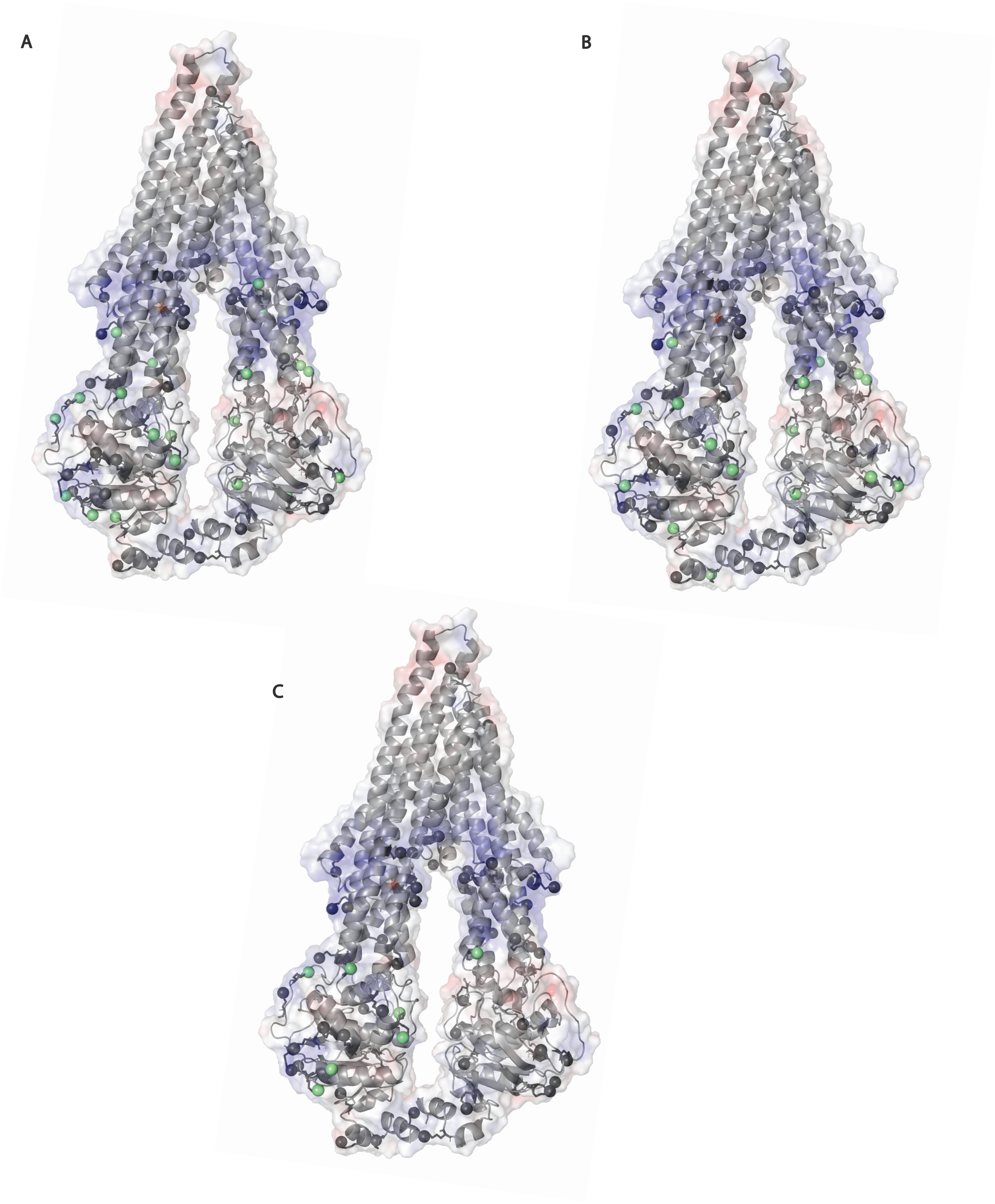

Figure S2. Mono-links of Pgp visualized on its tertiary structure, PDB 6qex. Mono-links on Pgp in the **(A)** on-bead DSSO, **(B)** the living cell DSSO and **(C)** living cell BS2Gd0/d4 samples.

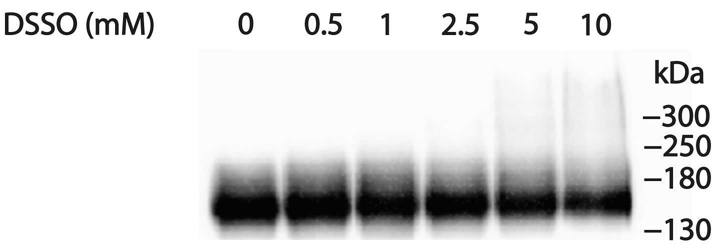

Figure S3. Western Blot analysis of Pgp cross-linked with different dilutions of DSSO. By adding increasing concentrations of DSSO, gradually more signal of Pgp appears at the higher molecular weight levels. No dimer formation of Pgp can be detected at concentrations of 0.5-10 mM DSSO.

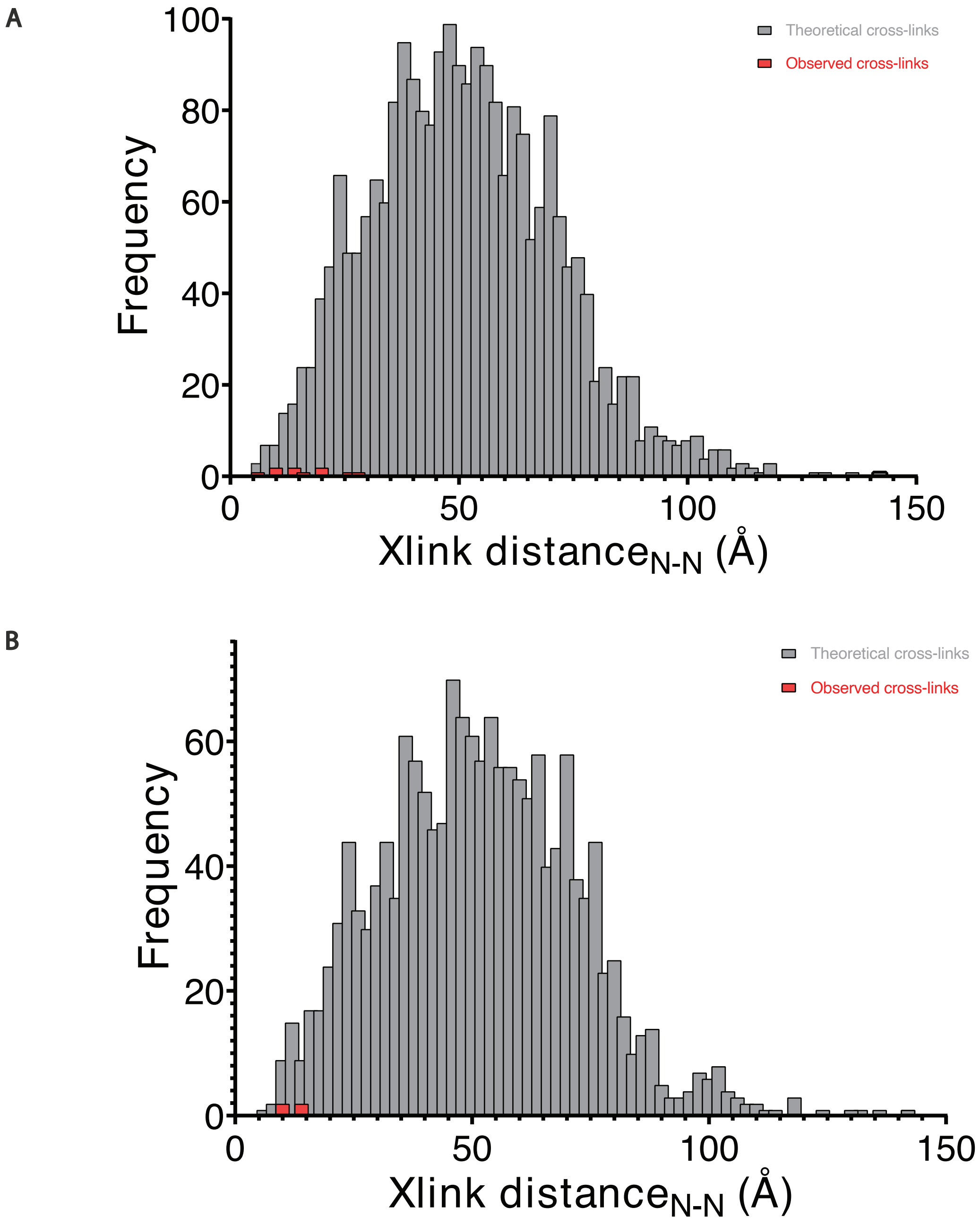

Figure S4. Distribution of distances between ε-amino groups of mono-linked lysine residues in the **(A)** on-bead DSSO and **(B)** living cell DSSO samples. All possible cross-links between the mono-linked sites are indicated in grey, and the observed cross-links by LC-MS/MS are red. All identified cross-links were between 5–30 Å which corroborates the validity of the MS results.

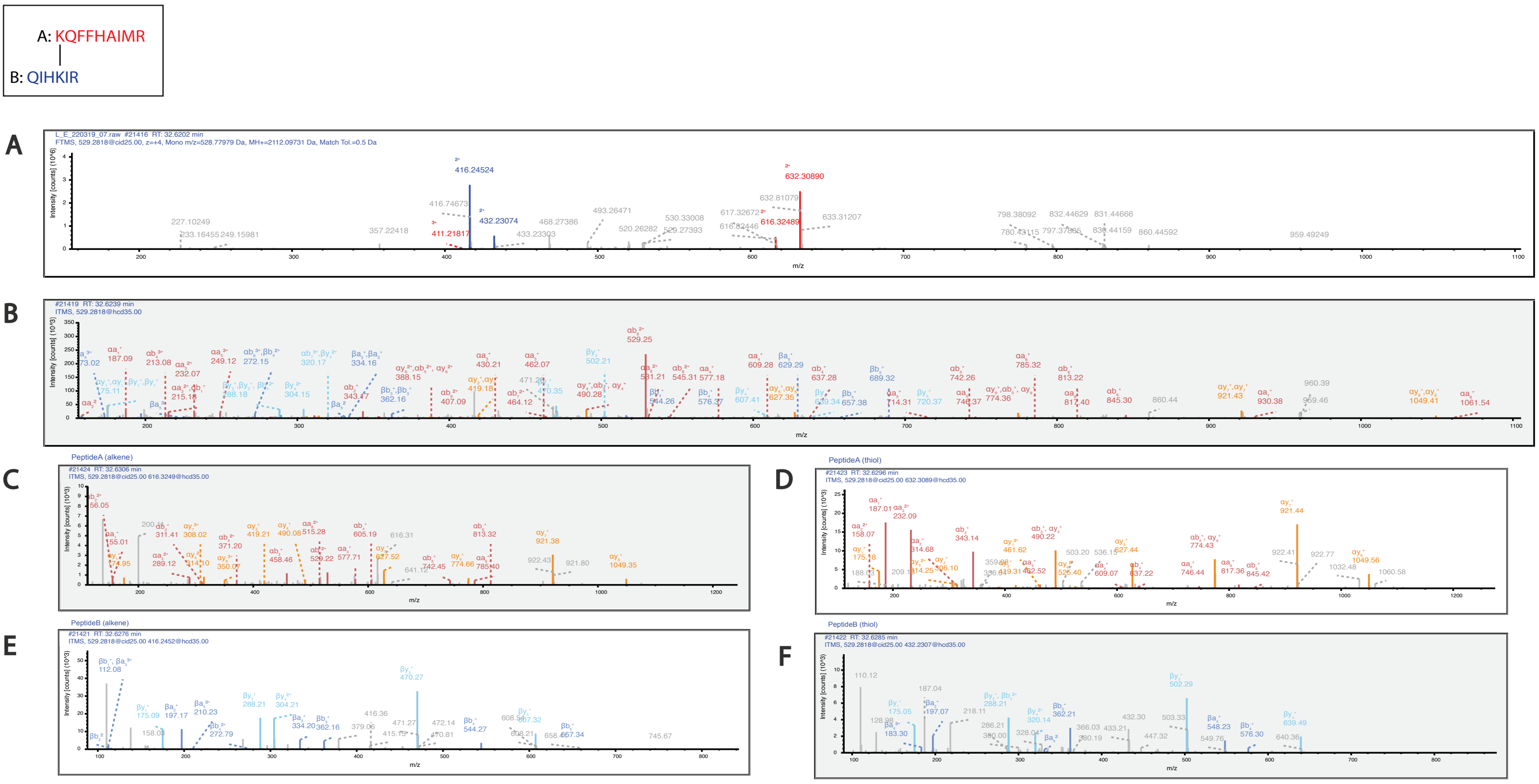

Figure 5. Pgp K146 is cross-linked to K149. **(A)** The MS2-CID spectrum is dominated by the diagnostic peptide pairs of the two cross-linked peptides formed upon fragmentation along the cross-linker. **(B)** The MS2-HCD spectrum contains additional fragments of the two peptides along the peptide backbone to assist identification. Fragments labeled red and orange belong to Peptide A while fragments labeled blue and light blue belong to Peptide B. **(C-F)** MS3-HCD spectra of the peptide pairs detected in MS2-CID confirm identity of Peptide A **(C, D)** and Peptide B **(E, F)**. Peptide A is **KQFFHAIMR** [149-157], Peptide B is **QIHKIR** [143-147] interpreted by XlinkX.

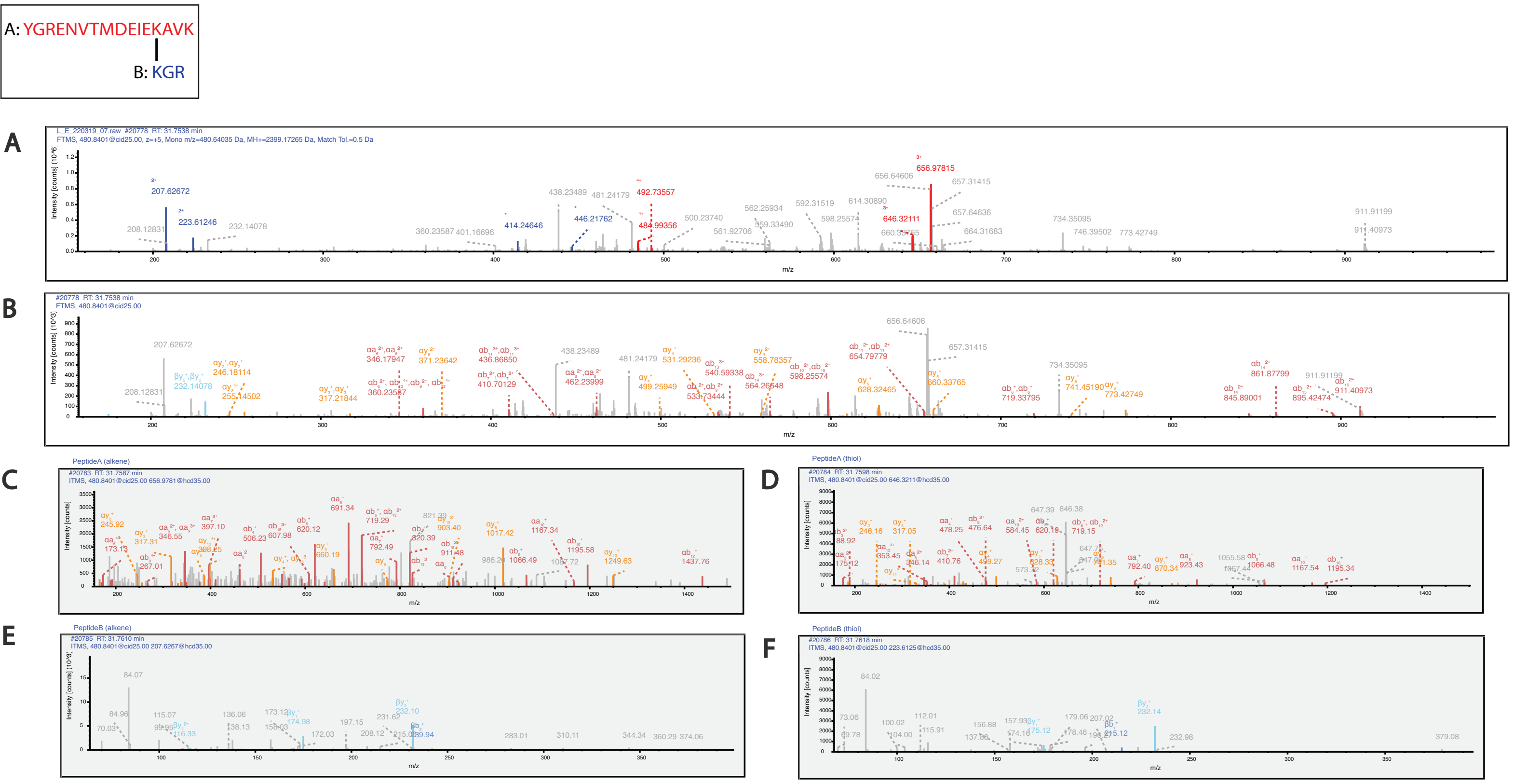

Figure S6. Pgp K502 is cross-linked to K578. **(A)** The MS2-CID spectrum is dominated by the diagnostic peptide pairs of the two cross-linked peptides formed upon fragmentation along the cross-linker. **(B)** The MS2-HCD spectrum contains additional fragments of the two peptides along the peptide backbone to assist identification. Fragments labeled red and orange belong to Peptide A while fragments labeled blue and light blue belong to Peptide B. **(C-F)** MS3-HCD spectra of the peptide pairs detected in MS2-CID confirm identity of Peptide A **(C, D)** and Peptide B **(E, F)**. Peptide A is **YGRENVTMDEIEKAVK** [490-505], Peptide B is **KGR** [578-580] interpreted by XlinkX.

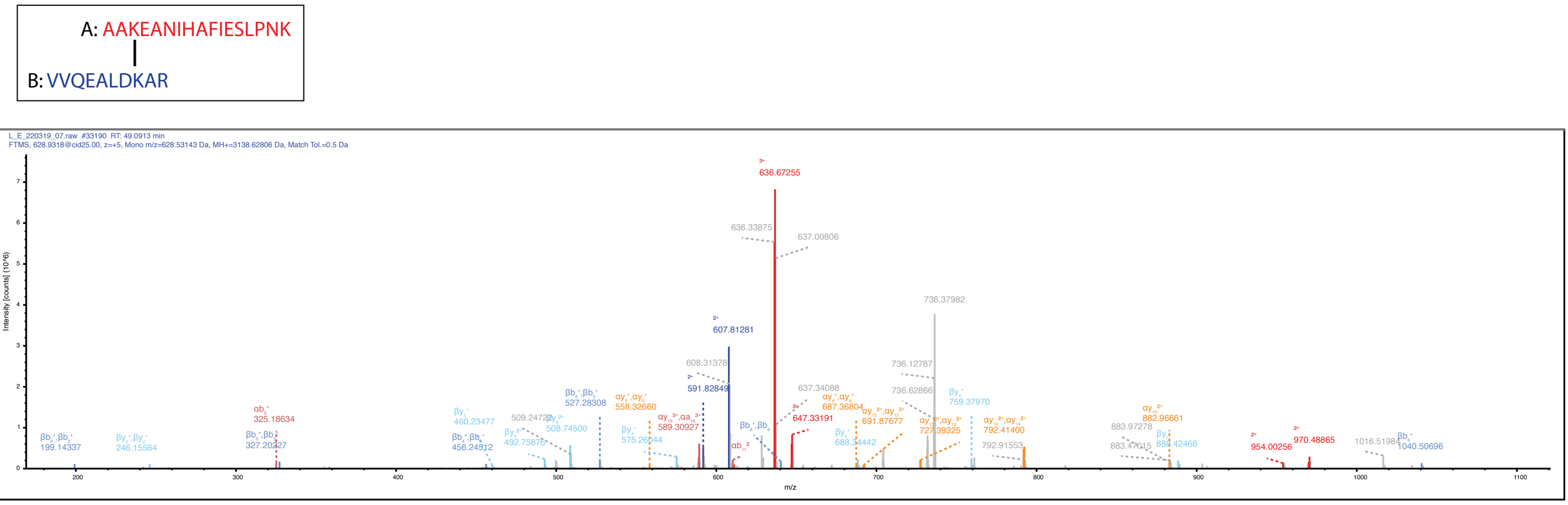

Figure S7. Pgp K1150 is cross-linked to K1220. The MS2-CID spectrum is dominated by the diagnostic peptide pairs of the two cross-linked peptides formed upon fragmentation along the cross-linker. The spectrum contains additional fragments of the two peptides along the peptide backbone to assist identification. Fragments labeled red and orange belong to Peptide A while fragments labeled blue and light blue belong to Peptide B. Peptide A is AAKEANIHAFIESLPNK [1148-1164], Peptide B is VVQEALDKAR [1213-1222] interpreted by XlinkX.

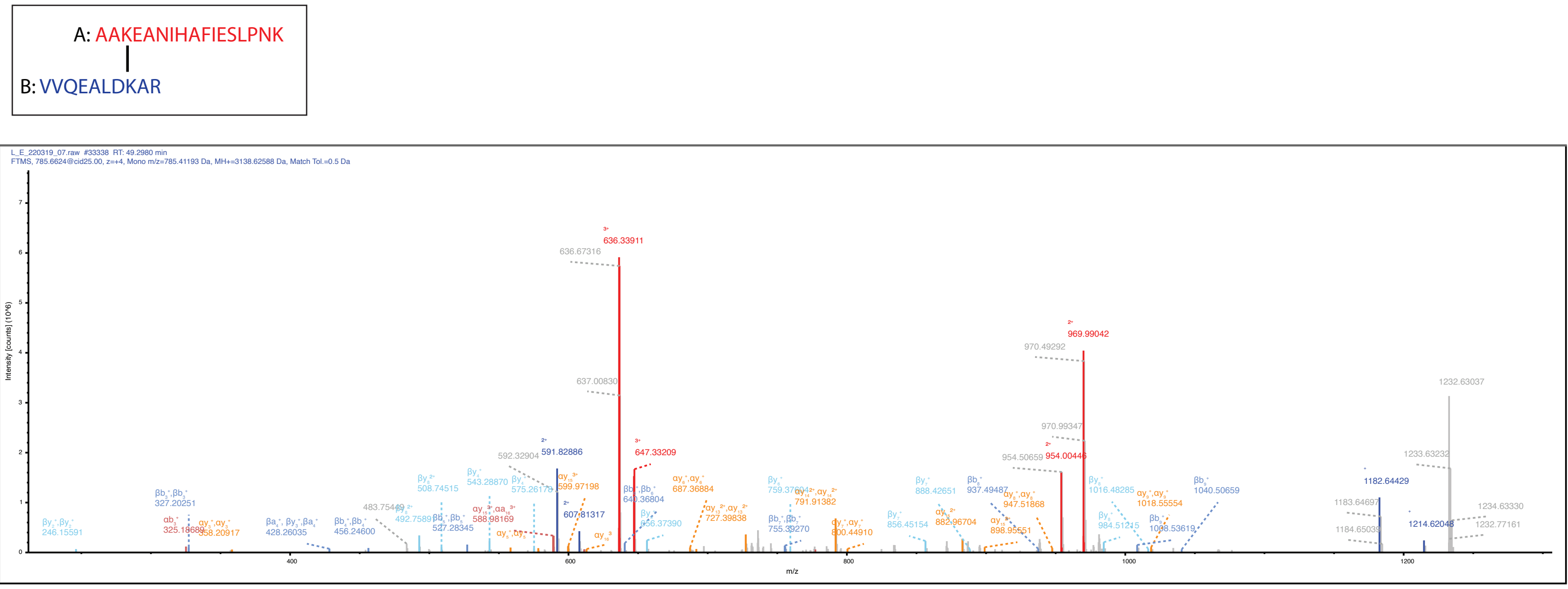

Figure S8. Pgp K1150 is cross-linked to K1220. The MS2-CID spectrum is dominated by the diagnostic peptide pairs of the two cross-linked peptides formed upon fragmentation along the cross-linker. The spectrum contains additional fragments of the two peptides along the peptide backbone to assist identification. Fragments labeled red and orange belong to Peptide A while fragments labeled blue and light blue belong to Peptide B. Peptide A is AAKEANIHAFIESLPNK [1148-1164], Peptide B is VVQEALDKAR [1213-1222] interpreted by XlinkX.

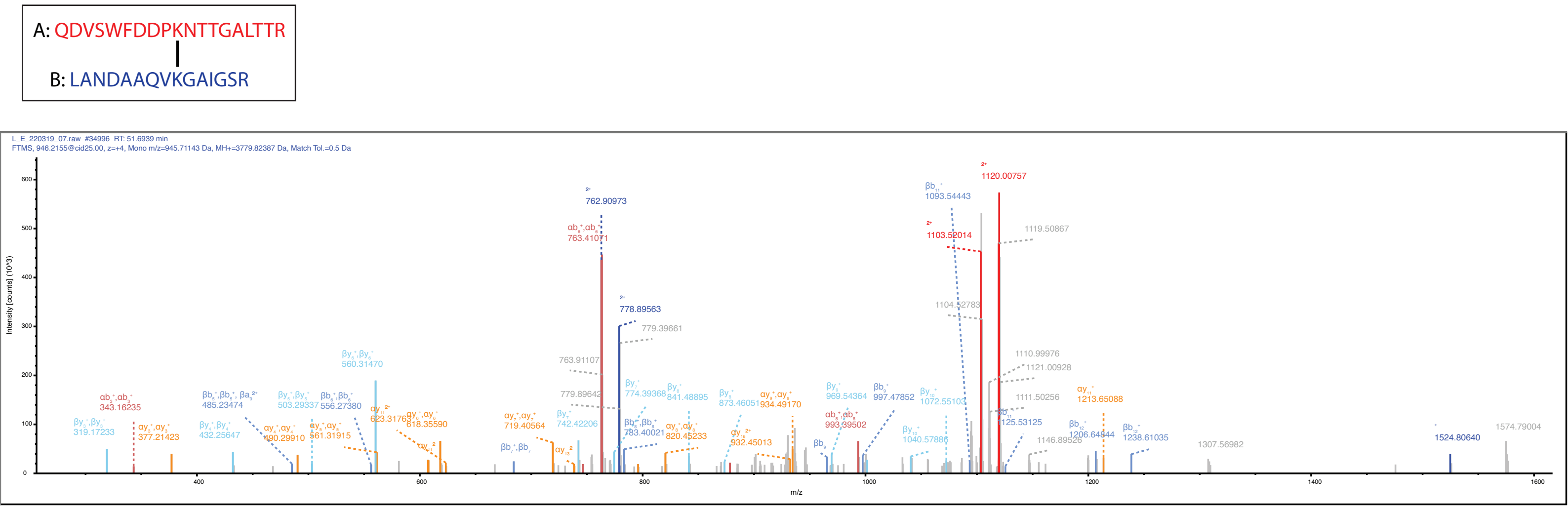

Figure S9. Pgp K808 is cross-linked to K826. The MS2-CID spectrum is dominated by the diagnostic peptide pairs of the two cross-linked peptides formed upon fragmentation along the cross-linker. The spectrum contains additional fragments of the two peptides along the peptide backbone to assist identification. Fragments labeled red and orange belong to Peptide A while fragments labeled blue and light blue belong to Peptide B. Peptide A is QDVSWFDDP**K**NTTGALTTR [799-817], Peptide B is LANDAAQV**K**GAIGSR [818-832] interpreted by XlinkX.

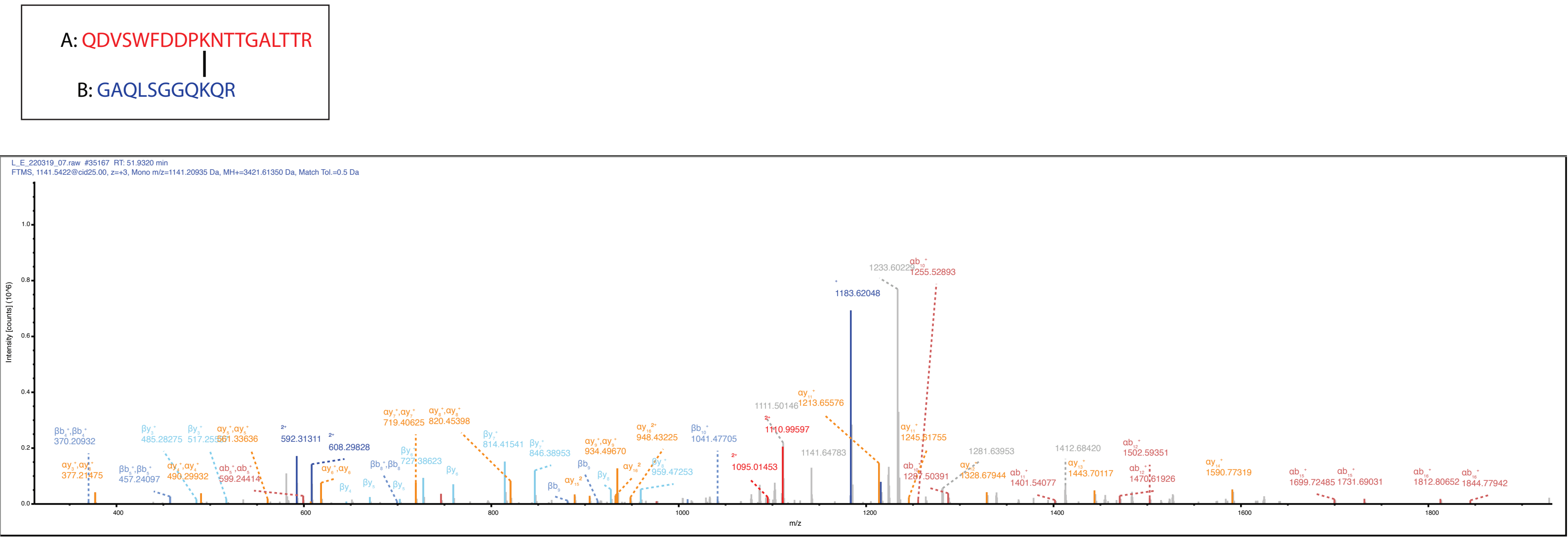

Figure S10. Pgp K536 is cross-linked to K808. The MS2-CID spectrum is dominated by the diagnostic peptide pairs of the two cross-linked peptides formed upon fragmentation along the cross-linker. The spectrum contains additional fragments of the two peptides along the peptide backbone to assist identification. Fragments labeled red and orange belong to Peptide A while fragments labeled blue and light blue belong to Peptide B. Peptide A is QDVSWFDDP**K**NTTGALTTR [799-817], Peptide B is GAQLSGGQ**K**QR [528-538] interpreted by XlinkX.

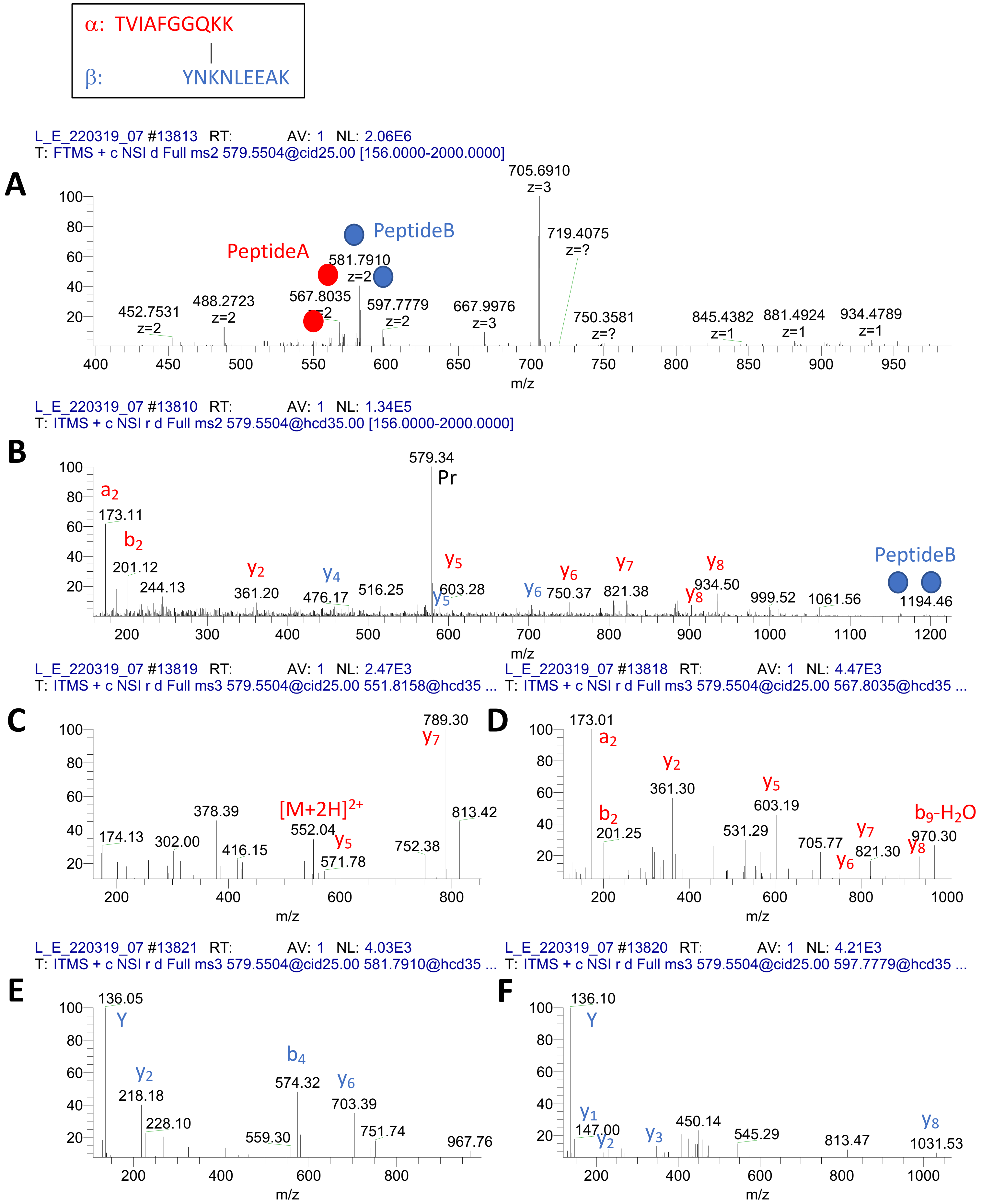

Figure S11. Pgp K271 is cross-linked to K279**.** The MS2-CID spectrum (**A**) displays the diagnostic peptide pairs of the two cross-linked peptides formed upon fragmentation along the cross-linker. Peptide A and B were identified from MS3-HCD spectra (**D** and **F**, respectively) by database search and further confirmed by fragments detected in the MS2-HCD spectrum (**B**). Peptide A is TVIAFGGQ***K***K [263-272], Peptide B is YN***K***NLEEAK [277-285]. Pr denotes unfragmented precursor ion in the MS2-HCD spectrum.

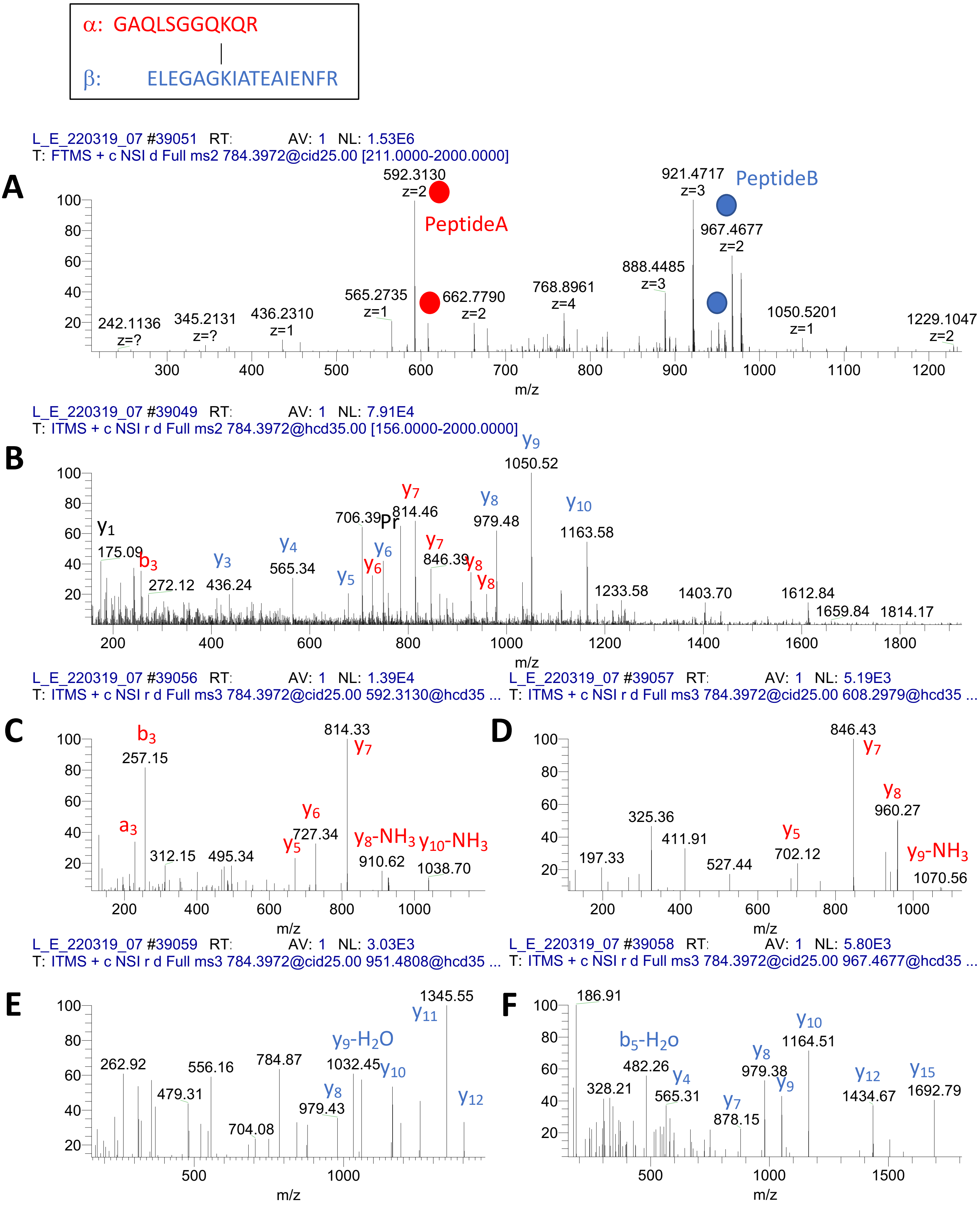

Figure S12. Pgp K536 is cross-linked to K895. The MS2-CID spectrum (**A**) displays the diagnostic peptide pairs of the two cross-linked peptides formed upon fragmentation along the cross-linker. Peptide A and B were identified from MS3-HCD spectra (**C-D** and **E-F**, respectively) by database search and further confirmed by fragments detected in the MS2-HCD spectrum (**B**). Peptide A is GAQLSGGQ***K***QR [528-538], Peptide B is ELEGAGKIATEAIENFR [889-905] which differs in an S893→A substitution to the canonical sequence in Swissprot. Pr denotes unfragmented precursor ion.

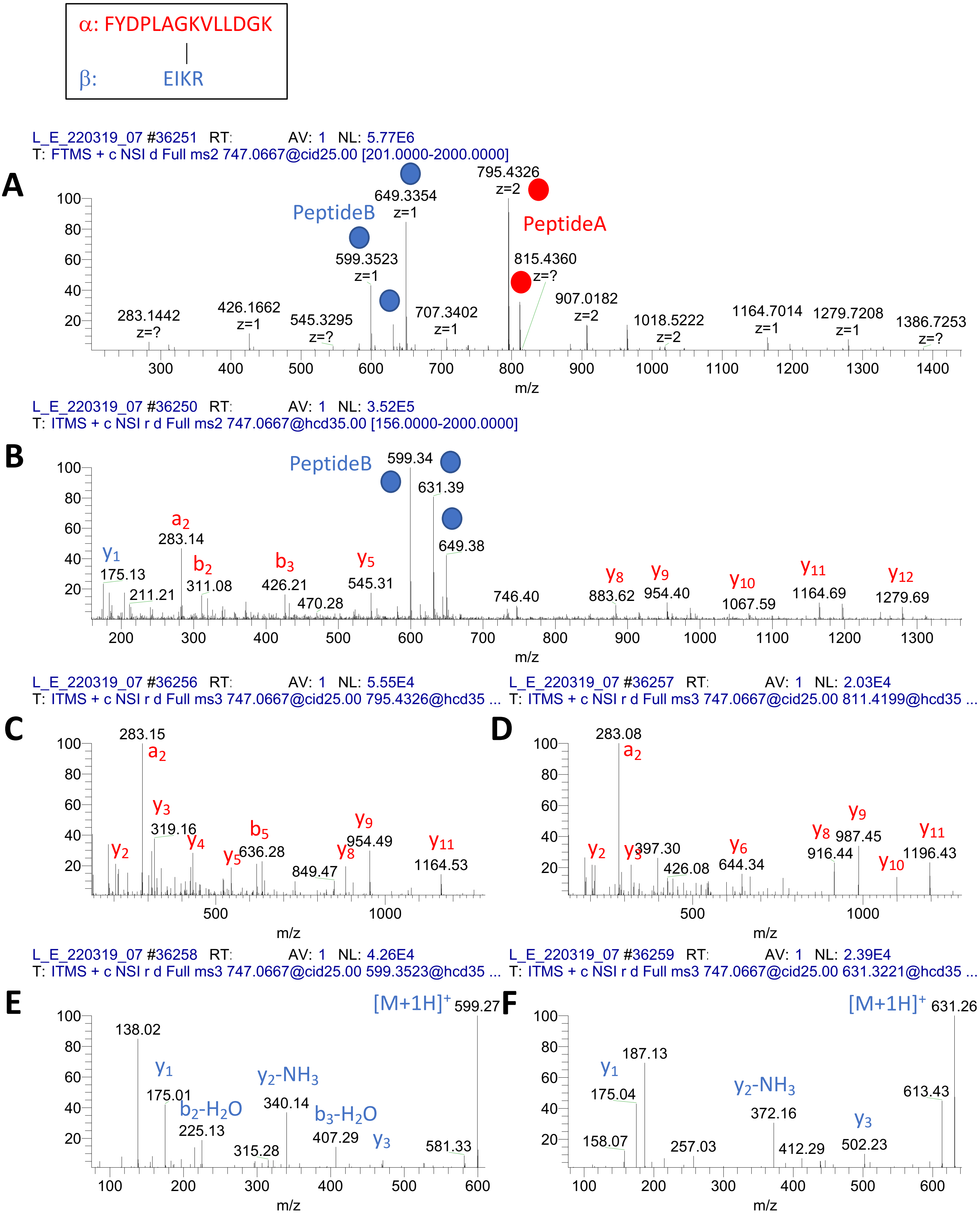

Figure S13. Pgp K1093 is cross-linked to K1102. The MS2-CID spectrum (**A**) displays the diagnostic peptide pairs of the two cross-linked peptides formed upon fragmentation along the cross-linker. Peptide A was identified from MS3-HCD spectra (**C, D**) by database search and further confirmed by fragments detected in the MS2-HCD spectrum (**B**). Peptide B was too short to be identified by database search, but its presence could be confirmed based on accurate mass of the precursor, the diagnostic peptide fragments in the MS2-CID and MS2-HCD spectra and fragments observed in the MS3-HCD spectra (**E, F**). Peptide A is FYDPLAG***K***VLLDGK [1086-1099], Peptide B is EI***K***R [1100-1103].

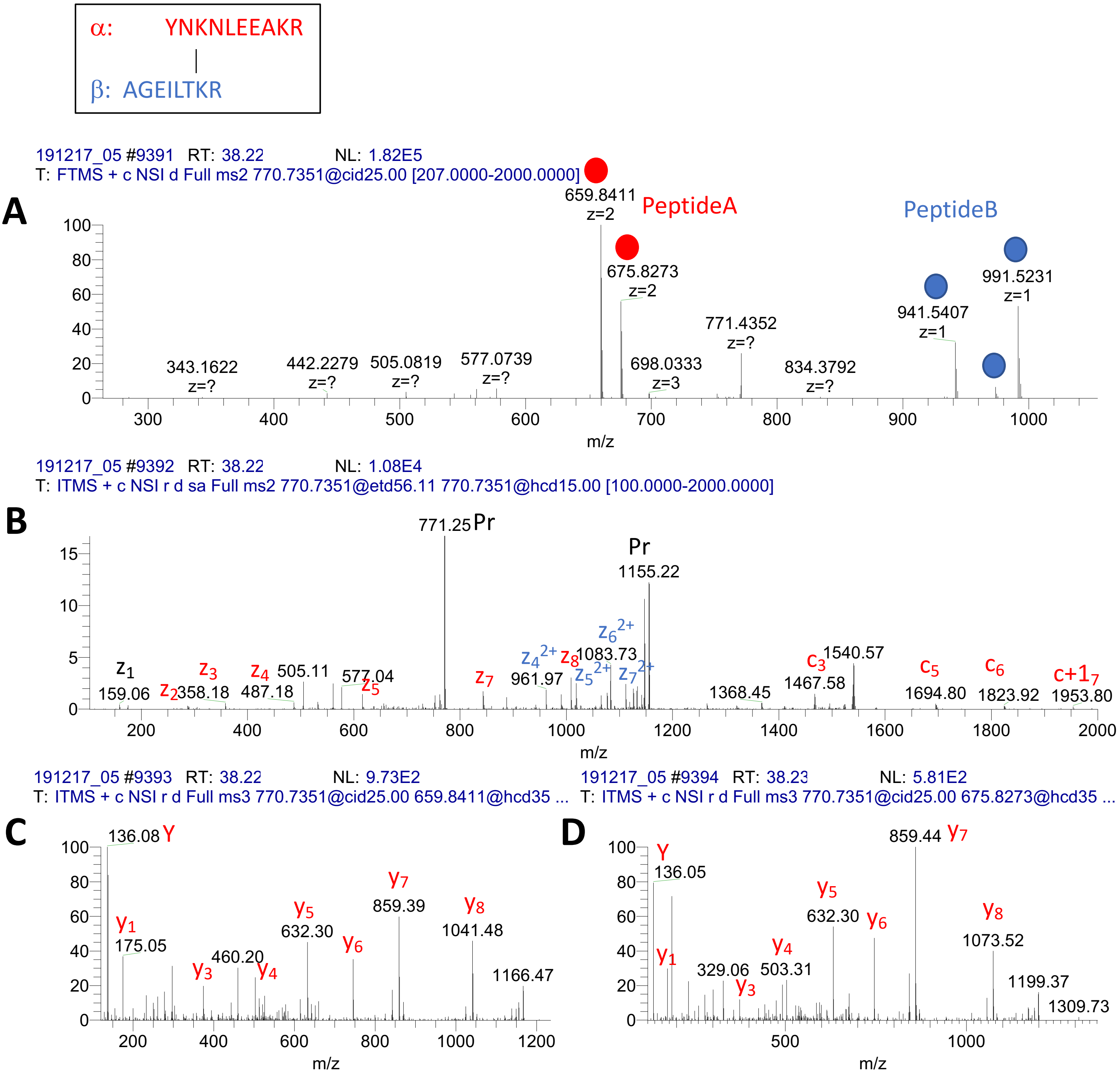

Figure S14. Pgp K279 is cross-linked to K786. The MS2-CID spectrum (**A**) displays the diagnostic peptide pairs of the two cross-linked peptides formed upon fragmentation along the cross-linker. Peptide A was identified from MS3-HCD spectra (**C, D**) by database search and further confirmed by fragments detected in the MS2-EThcD spectrum (**B**). Peptide B was identified based on accurate mass of the precursor, the diagnostic peptide fragments in the MS2-CID spectrum and additional fragments observed in the MS2-EThcD. Peptide A is YN***K***NLEEAKR [277-286], Peptide B is AGEILT***K***R [780-787]. Pr denotes unfragmented precursor ion.

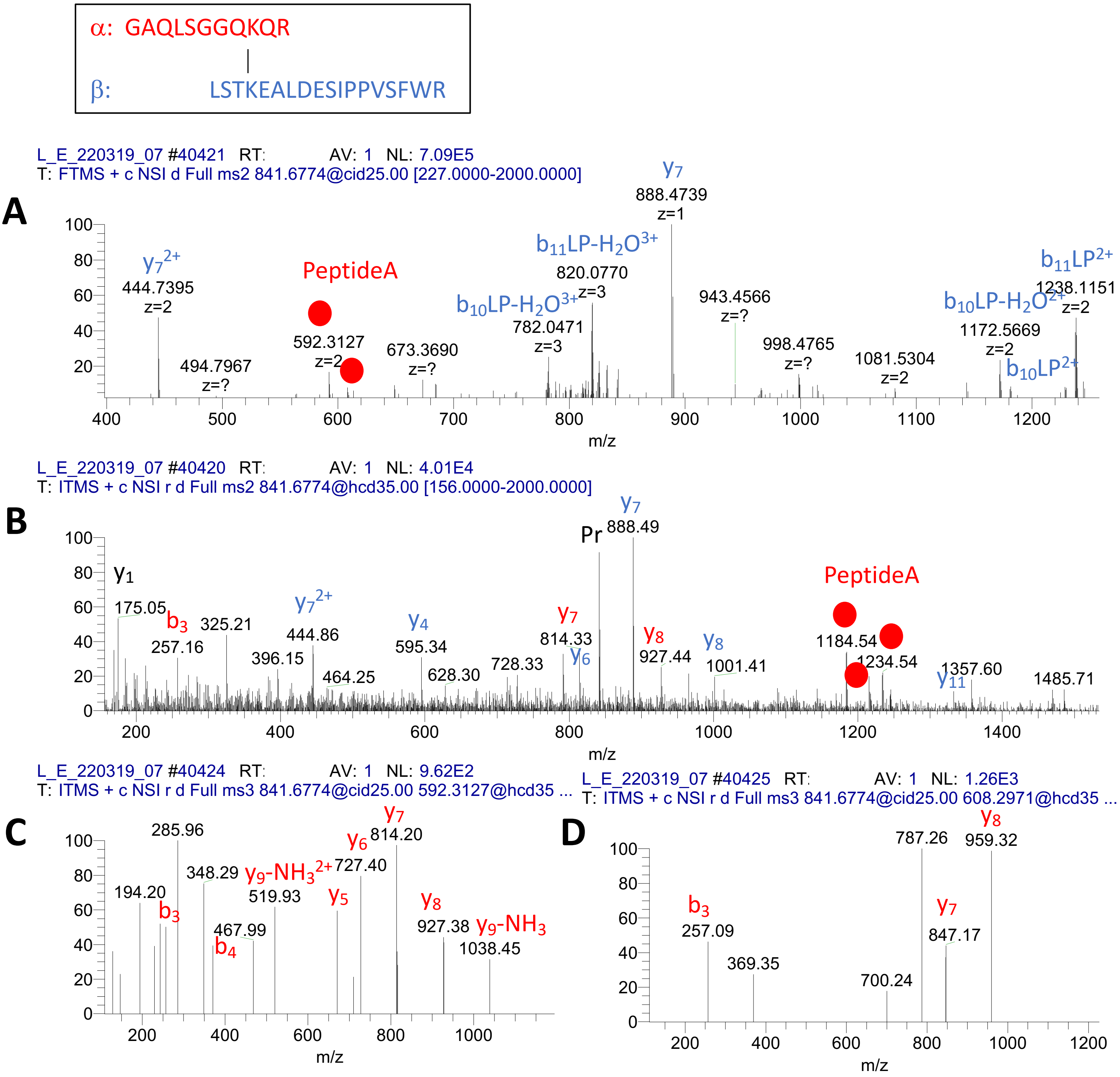

Figure S15. Pgp K536 is potentially cross-linked to K685. The MS2-CID spectrum (**A**) displays the diagnostic peptide pair only for Peptide A which was identified as GAQLSGGQ***K***QR [528-538] from an MS3-HCD spectrum (**C**) by database search. Based on accurate mass of the precursor, Peptide A is potentially crosslinked to LST***K***EALDESIPPVSFWR [682-699]. Additional fragments detected in the MS2-CID (**A**) and MS2-HCD (**B**) spectra strengthen this assumption. The peptide fragments that are most abundantly detected are explained with cleavage at the N-terminus of a proline in the putative cross-linked peptide. Such bonds are generally very fragile upon CID/HCD fragmentation which is a possible reason why the DSSO specific fragmentation is not detected for this peptide. Pr in the MS2-HCD spectrum denotes unfragmented precursor ion.

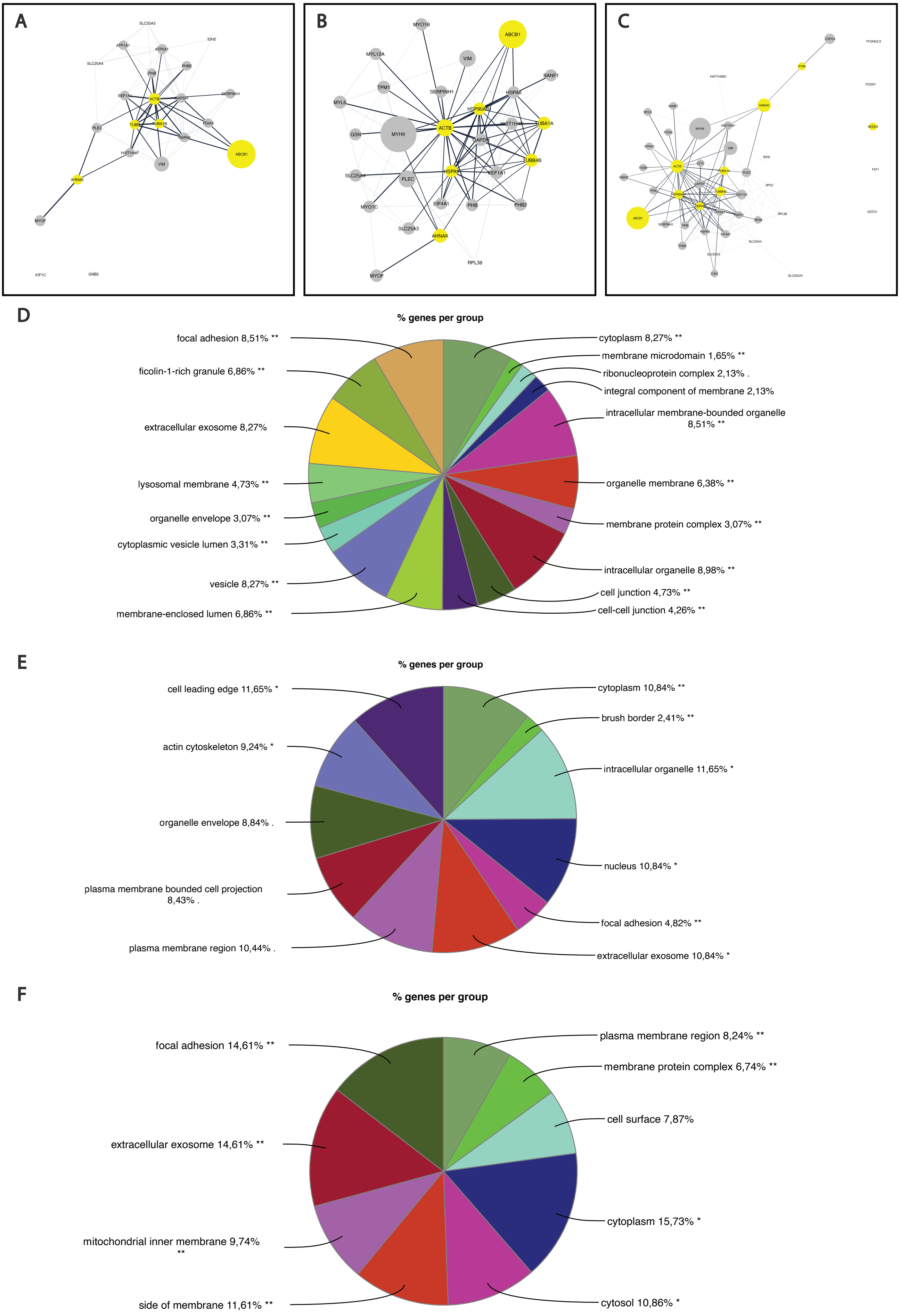

Figure S16. Differences between 15D3 and UIC2 IP enrichments. Protein interaction networks of **(A)** living cell DSSO approach, enriched with UIC2 mAb, **(B)** living cell BS2Gd0/d4 approach, enriched with UIC2 mAb and **(C)** living cell BS2Gd0/d4 approach, enriched with 15D3 mAb. Semiquantitative abundance of individual proteins is depicted by the size of the nodes based on spectral counts (SPCs). Interactions were gathered and depicted using the STRING data-base and Cytoscape software. Cellular component distribution of **(D)** living cell DSSO approach, enriched with UIC2 mAb, **(E)** living cell BS2Gd0/d4 approach, enriched with UIC2 mAb and **(F)** living cell BS2Gd0/d4 approach, enriched with 15D3 mAb. Cellular component distribution was analyzed with the ClueGO app within Cytoscape.
